# Real Life Data on Patient-Reported Outcomes and Neuro-Cognitive Functioning of Lung Cancer Patients: The PRO-Long Study

**DOI:** 10.3389/fonc.2021.685605

**Published:** 2021-06-17

**Authors:** Lotte Van Der Weijst, Veerle Surmont, Wim Schrauwen, Yolande Lievens

**Affiliations:** ^1^ Department of Radiation Oncology, Ghent University Hospital, Ghent, Belgium; ^2^ Department of Respiratory Medicine, Ghent University Hospital, Ghent, Belgium; ^3^ Department of Medical Psychology, Ghent University Hospital, Ghent, Belgium

**Keywords:** health-related quality of life, non-small cell lung cancer, neuro-cognition, real-life data, patient-reported outcome, toxicity

## Abstract

**Introduction:**

This report investigates the impact of systemic treatments (chemotherapy or immunotherapy) with(out) loco-regional radiotherapy, on HRQoL, toxicity and neurocognitive functioning (NCF) in locally advanced and metastatic non-small cell lung cancer patients enrolled in the PRO-Long study.

**Materials and Methods:**

Data on patient-reported HRQoL and fourteen toxicities was collected, while NCF was tested, up to one-year post-treatment. HRQoL was assessed using the European Organisation for Research and Treatment of Cancer QLQ-C30. Lung cancer, treatment and neuro-psychological related toxicities were scored with the Patient-Reported Outcomes version of the Common Terminology Criteria for Adverse Events. NCF was evaluated with six neurocognitive tests. Mixed model analyses were conducted to determine statistical significance (p = .01). Meaningful clinical important differences (MCIDs) were applied for changes in HRQoL and NCF data, while toxicities were compared to baseline values.

**Results:**

In total, 50 patients were enrolled. Overall HRQoL (p = .357) nor its domains (physical, p = .643; role, p = .069; emotional, p = .254; cognitive, p = 494; social, p = .735) changed significantly over time. Meaningful improvements in overall HRQoL were seen in 22, 38 and 39% and deteriorations in 22, 5 and 28% of patients at 2–3, 6 and 12 months respectively post-treatment. Overall toxicity (p = .007), lack of appetite (p = .001), nausea (p = .004) and dysphagia (p = .000) significantly decreased over time. Treatment caused acute toxicity, such as dyspnoea (45%) and memory problems (42%), but also alleviated pre-existing symptoms, including lack of appetite (32%), anxiety (29%) and depression (28%) at 2/3 months. The NCF domains of visual memory (p = .000) and cognitive processing speed (p = .000) showed significant improvements over time. In terms of MCIDs, at 2–3 months (18%) and 6 months (15%), verbal memory was particularly impacted; at 12 months, visual memory (18%) and executive function (18%) deteriorated primarily.

**Conclusion:**

The results suggest that therapy has no significant negative impact on overall HRQoL, its domains, and NCF. About one-third of patients reported a meaningful improved HRQoL at 1 year post-treatment. Treatment caused toxicity, but also alleviated pre-existing symptoms.

## Introduction

Lung cancer is the leading cause of cancer death worldwide (1). Roughly 85% of all newly diagnosed lung cancer patients presents with non-small cell lung cancer (NSCLC), of which the majority is locally-advanced (LA-) or metastatic disease (2). Whereas for LA-NSCLC a wide variety of treatment combinations is available with the intent to cure, the main aim of treatment for metastatic patients is to maintain or increase quality and quantity of life (3).

Rapid developments in treatment have enhanced survival outcomes in both LA- and metastatic NSCLC, but in general survival remains poor. Standard and novel therapies and their combinations may have different life-impacting adverse events (4, 5). Therefore, the focus on the effect of treatment on wellbeing and functioning becomes increasingly important. It has been shown that lung cancer and its associated treatment indeed impacts health-related quality of life (HRQoL), they bring along several acute and late toxicities and may negatively affect neurocognitive functioning (6–9).

Patient-reported outcomes (PROs), such as HRQoL or toxicity, directly reflect any aspect of patients’ perspective concerning health, burden of disease and treatment, feelings and functioning without interpretation by healthcare professionals or anyone else (10). It has been increasingly acknowledged that patient-reported outcome measurements (PROMs) are valid and reliable tools to be used in clinical trials and daily clinical practice to capture patients’ wellbeing (10, 11). PROMs promote patient-centred care, aid in decision-making, facilitate early detection of serious events, improve patient-clinical communication and support symptom management. PROMs have gained popularity in clinical trials, but although there are multiple initiatives to embed them in daily clinical practice, data so far remain limited (12).

Besides the impact on PROs, both the treatment and the cancer itself may also induce cognitive impairments, which are a widespread problem for cancer patients. The term ‘chemobrain’ has emerged to describe the experiences of chemotherapy-related impaired cognition, particularly memory and concentration problems. However, contradictions exist on whether chemotherapy by itself affects cognition (13, 14). Radiation-induced cognitive decline may also occur, but has especially been documented in patients receiving cranial radiotherapy, with limited data obtained from patients being treated for loco-regional disease (15). Similarly, limited research, none in the lung cancer population, has been conducted on the impact of immunotherapy with(out) chemotherapy on NCF (16). Cognitive impairment in cancer patients may influence HRQoL, hence, it is important to be aware of the impact of treatment on cognitive functioning to maintain patients’ wellbeing (6).

We herewith report the results of the longitudinal PRO-Long study, collecting PROs and NCF in the real-world setting of a lung cancer cohort, of which the objectives were two-fold (1): examine patient-reported HRQoL and toxicity in LA- and metastatic NSCLC patients receiving systemic therapy with(out) loco-regional radiotherapy over time (2); explore neurocognitive functioning in this patient population.

We hypothesized that in this real-world patient population, patients could experience improved overall HRQoL over time, despite expected acute and long-term toxicities, and wanted to explore the evolution in NCF. We also hypothesized that changes over time may be related to the disease stage, and the herewith related baseline patient characteristics and treatment approaches.

## Materials and Methods

### Patient Population

Patients with LA-NSCLC receiving loco-regional radiotherapy and/or chemotherapy and metastatic NSCLC patients under first-line chemotherapy and/or immunotherapy or second-line immunotherapy at Ghent University Hospital (GUH), Belgium, were included in this prospective, observational cohort study. Only patients receiving the entire treatment and follow-up at GUH were invited to participate. The study was approved by GUH’s ethical committee. All patients gave written informed consent prior to participating in the study.

### Data Collection

Patient characteristics, such as Eastern Cooperative Oncology Group (ECOG)/World Health Organisation (WHO) performance status, smoking and relationship status and highest obtained education, and tumour characteristics were collected at baseline, whereas treatment specifics were obtained at the end of therapy.

PROMs on HRQoL and toxicity data were collected directly by the patient through paper questionnaires. HRQoL was measured with the European Organisation for Research and Treatment of Cancer (EORTC) Quality of Life Questionnaire Core 30 items (QLQ-C30) and the EORTC Quality of Life Questionnaire Lung Cancer 13 items (QLQ-LC13) (17–19). The QLQ-C30 measures five functional domains (physical, role, emotional, cognitive, and social), nine cancer symptoms and treatment-associated side-effects, global health and quality of life. Similarly, the QLQ-LC13 assesses lung cancer-specific symptoms and adverse events.

The toxicity PROMs were scored with fourteen items related to lung cancer (loss of appetite, pain, fatigue, dyspnoea, cough), therapy (vomiting, nausea, dysphagia, diarrhoea, constipation) and neuro-psychological problems (anxiety, depression, memory problems and concentration problems) of the PRO-CTCAE (20).

The PROM captures subjective cognitive functioning. To test objective cognitive performance, six neurocognitive tests (Rey Complex Figure Test and Recognition Trial (ROFL), Hopkins Verbal Learning Test-Revised (HVLT-R), Trail Making Test (TMT), Controlled Oral Word Association Test (COWA), Wechsler Adult Intelligence Scale (WAIS)–Digit Span and Stroop test) were administered testing immediate and delayed memory, recognition, cognitive processing speed, executive functioning, verbal fluency, working memory and attention.

To limit patient burden, PROMs and NCF tests were only administered when patients came to the hospital for follow-up consultation. Due to the fact that standard follow-up differs in our hospital between stages and to decrease patient burden, different time points for LA- and metastatic NSCLC were collected.

**Figure 1:**
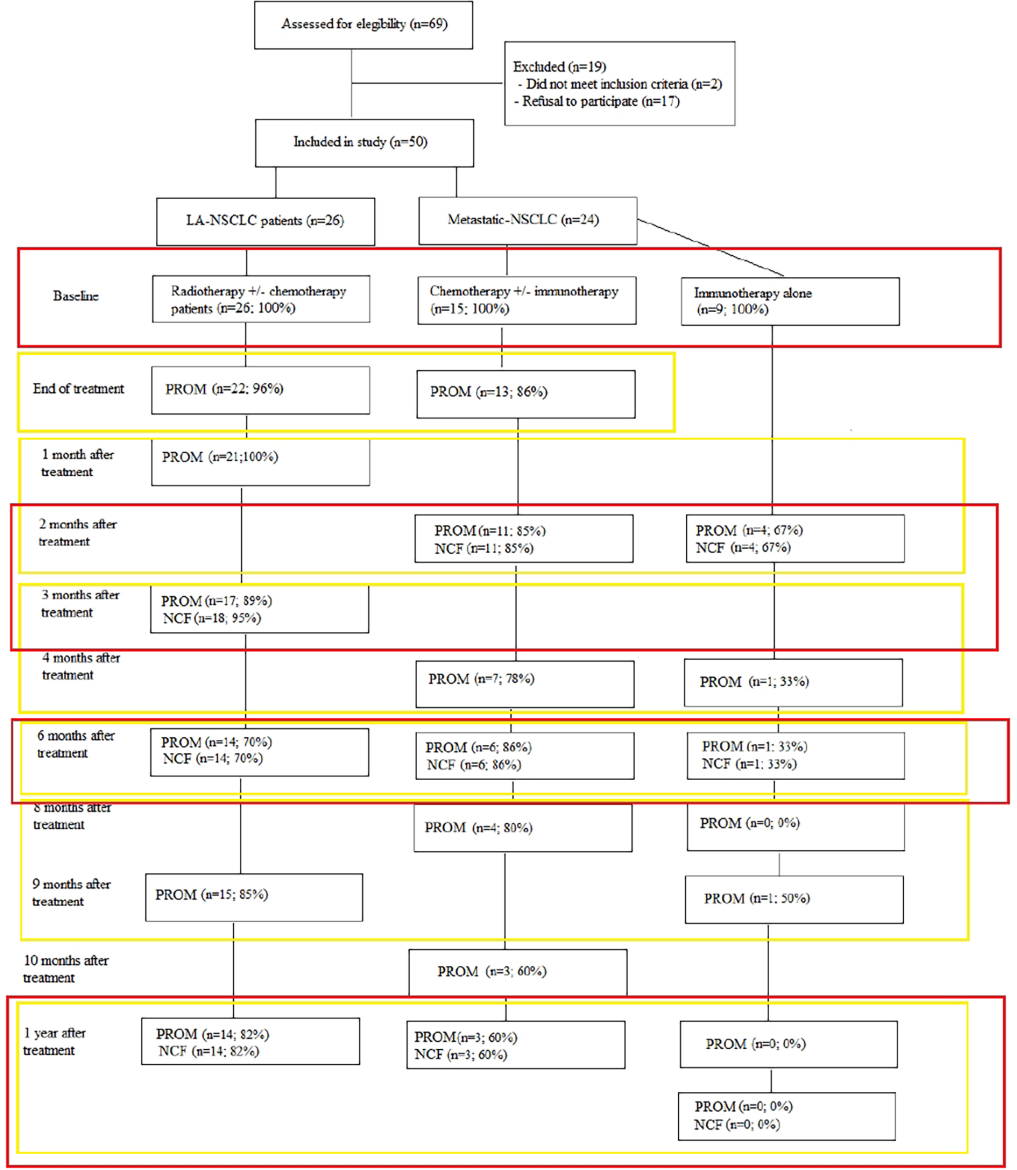
Overview of data collected per time point and data handling strategy. Red rectangles denote the data that were analysed together for the changes compared to baseline, the yellow rectangles show the data analysed together in the mixed model analyses.

LA-NSCLC patients were asked to fill out PROMs pre-treatment, at end of treatment, 1, 3, 6, 9 and 12 months post-treatment. Metastatic patients receiving chemotherapy alone or combined with immunotherapy received PROMs pre-treatment, at end of chemotherapy, 2, 4, 6, 8, 10 and 12 months post-treatment. For those receiving immunotherapy alone, no end of treatment was defined. Therefore, they completed PROMs pre-treatment, 2, 4, 6, 8, 10 and 12 months after start of immunotherapy. Cognitive tests were administered at baseline and 2–3, 6 and 12 months post-treatment in LA-NSCLC and metastatic patients receiving chemo ± immunotherapy. Patients receiving first- or second line immunotherapy performed NCF testing at baseline, 2, 6 and 12 months after start of the treatment (**Figure 1**). Study follow-up was terminated when patients received systemic treatment for progressive disease or brain irradiation.

### Data Handling and Statistical Analysis

HRQoL data of the EORTC QLQ-C30 and LC13 was calculated based on the scoring manual of the EORTC (18). A raw score was calculated as the average of the items of each scale (symptoms, functional and global quality of life). A linear transformation was used to standardize the raw scores, ranging from 0 to 100. Higher scores in the functional scales, global quality of life and health status indicate better functioning, whereas higher scores in the symptom scales indicate higher symptom burdens. HRQoL data was considered missing if at least half of the items of the EORTC QLQ-C30 and QLQ-LC13 questionnaires were missing. Only data on the different domains and on overall HRQoL were included in these analyses. A 10 point change in score within a patient over time was considered the threshold of a minimally clinical important difference (MCID) (10).

Toxicities were calculated by subtracting baseline scores from subsequent treatment-related toxicities. To estimate overall toxicity, the Standardized Total Average Toxicity (STAT) score was used (21). The score was calculated based on the mean of z-scores of each toxicity derived from the CTCAE score for each patient and time point. The STAT score summarizes the various CTCAE outcomes into a single value and represents an overall measure of the individuals’ overall toxicity burden. STAT defines whether an individual patient experiences more or less toxicity than the mean of the population, higher STAT scores indicating more toxicity. In the mixed model analyses, only toxicities worsening from baseline were included.

For NCF, a change of ≥1.0 standard deviation (SD) between baseline and each consecutive time point was considered a cut-off demarcating a mild cognitive impairment or improvement and is therefore considered a MCID. In the mixed model analyses, only cognitive impairments were included.

Explorative analyses on PROMs and NCF were done for the different disease stages (LA- and metastatic).

Due to the different time-points of data collection, changes over time and the associations between aforementioned concepts were calculated for 2–3, 6 and 12 m, compared to baseline. Compliance levels were calculated by dividing the number of PROMs and NCF tests received by the number expected at each assessment point.

Multilevel analyses conducted to evaluate whether there was a significant change in PROM and NCF scores over time included all time points (**Figure 1**).

Analyses were performed with SPSS version 26.0. Descriptive statistics were used for the population characteristics and the changes from baseline. For the analyses, the statistical significance of changes over time, a mixed models method with a compound symmetry structure was used as suggested by Hamel et al. (22). This analysing technique was applied because of the hierarchical structure of the data, the ability to account for repeated measures across participants and for missing data, a common phenomenon in the lung cancer population (7). The level of statistical significance was set at p = 0.01 to correct for multiple comparisons and to adjust for a level I error.

## Results

### Patient Characteristics

In total, 67 patients were asked to take part in the study, of which 50 agreed to participate and accrued from January 2016 through December 2018. The 17 patients declining participation mainly did so because of the anticipated mental burden of participation. The introduction of clinical trials on first-line immunotherapy impeded the recruitment of metastatic NSCLC patients. Therefore, the inclusion criteria were changed to include patients receiving first and second-line immunotherapy.

The majority of included patients were male (64%), with minor symptoms (ECOG/WHO performance status of 1) yet important cardio-vascular and pulmonary co-morbidities.

There was a preponderance of stage III disease (n = 26; 56%) for which concurrent chemo-radiotherapy (n = 19; 73%) was the most frequent treatment approach. In stage IV (n = 24; 46%) chemotherapy alone (n = 12; 50%) was received most often. Of those still participating at 1 year, a higher proportion of males remained (n = 12; 71%), especially those with better performance and higher education at baseline.

Similarly, those with better physical (87.72 *vs* 79.86) and role functioning (79.82 *vs* 73.12), as well as experiencing less general pain (4.7 *vs* 20.0) at baseline tended to have a higher chance of surviving 1 year. Even if missing data was frequently encountered, overall compliance was high with 100% at baseline to 77% at 1 year post-treatment (i.e. 17 out of 22 patients alive without progression) for both PROs and NCF. In total, 18 patients were still alive at 1 year, however, one patient decided to not take part in the NCF tests, but filled out the PROM and one patient took part in the NCF tests, but not in the PROM. Follow-up was mostly terminated due to death and health deterioration. At one year, no first nor second-line immunotherapy alone patients were still participating. Particularly, mainly LA-NSCLC patients continued to participate until 1 year after treatment, whereas of those with metastatic NSCLC, only three out of 24 were still participating at 1 year.

Full details on patient, tumour and treatment characteristics and baseline HRQoL data of all participants and those still participating at 1 year are shown in **Table 1**. **Figure 1** provides an overview of numbers and of compliance in percentages to the PROMs and NCF tests.

**Table 1:** Patient, tumor, treatment and HRQoL characteristics at baseline and for 1-year survivors.

	Overall population (n = 50)	1-year survivors (n = 18)
**Patient characteristics**		
Male—n (%)	32 (64)	12 (70.5)
Age—mean (±SD)	63.4 (8.86)	60.6 (8.8)
WHO Performance Status—n (%) 0 1 2	15 (30) 33 (66) 2 (4)	8 (44) 10 (56)
Comorbidities—n (%) COPD Cardio-vascular disease Hypertension Arrhythmias Other cardio-vascular disease Depression	30 (60) 21 (42) 19 (38) 3 (6) 3 (6) 8 (16)	11 (61) 7 (39) 7 (39) 4 (22)
BMI—n (%) Underweight (<18.5) Normal (18.5 – 24.9) Overweight (25 – 29.9) Obese (>30)	3 (6) 24 (48) 16 (32) 7 (14)	1 (6) 9 (50) 4 (22) 4 (22)
Smoking status—n (%) Never smoker Ex-smoker before cancer diagnosis Ex-smoker, since cancer diagnosis Current	5 (10) 23 (46) 7 (14) 15 (30)	4 (22) 4 (22) 9 (50) 1 (6)
Education—n (%) Primary school Secondary school Higher education University	6 (12) 29 (58) 10 (20) 5 (10)	9 (50) 5 (28) 4 (22)
Relationship status—n (%) In relationship Single	42 (84) 8 (16)	14 (78) 4 (22)
Children—n (%) Yes	44 (88)	14 (78)
Employment status—n (%) Currently employed Unemployed Retired	6 (12) 21 (42) 23 (46)	4 (22) 10 (56) 4 (22)
**Tumor characteristics**		
Stage—n (%) III IV	26 (54) 24 (46)	16 (89) 2 (11)
Histology—n (%) Adenocarcinoma Squamous-cell carcinoma Neuroendocrine carcinoma Undifferentiated	34 (68) 13 (26) 2 (4) 1 (2)	15 (83) 2 (11) 1 (6)
**Treatment characteristics**—n (%)		
Treatment modality Concurrent chemo-radiotherapy Sequential chemo-radiotherapy Radiotherapy alone Chemotherapy alone Chemo-immunotherapy Immunotherapy (1st line) Immunotherapy (2nd line)	19 (38) 5 (10) 4 (8) 12 (24) 1 (2) 6 (12) 3 (6)	13 (71) 1 (6) 1 (6) 1 (6) 2 (11)
Therapy response Partial response Stable disease Progressive disease Unknown	19 (38) 11 (22) 15 (30) 5 (10)	12 (67) 5 (28) 0 (0) 1 (5)
**HRQoL characteristics, average scores**		
QLQ-C30 scores Physical functioning Role functioning Emotional functioning Cognitive functioning Social functioning Fatigue Nausea and vomiting Pain Dyspnoea Insomnia Appetite loss Constipation Diarrhoea Financial difficulties	79.86 73.12 76.53 82.31 84.01 29.02 6.12 25.51 27.89 28.57 16.33 10.20 4.86 10.34	87.72 79.82 74.54 81.58 84.21 25.15 2.63 20.18 24.56 33.33 15.79 7.02 1.75 9.52
LC13 scores Cough Haemoptysis Dyspnoea Sore mouth Dysphagia Peripheral neuropathy Alopecia Pain in chest Pain in arm or shoulder Pain in other parts	38.19 5.55 22.91 0.69 5.55 6.25 6.94 18.06 17.36 20.00	33.3 4.17 19.44 0.0 2.08 4.17 6.25 16.67 25.0 4.76

Whether a patient is scored having a co-morbidity is based on the prescribed medication for the condition in the electronic patient records.

### HRQoL

**Figure 2:**
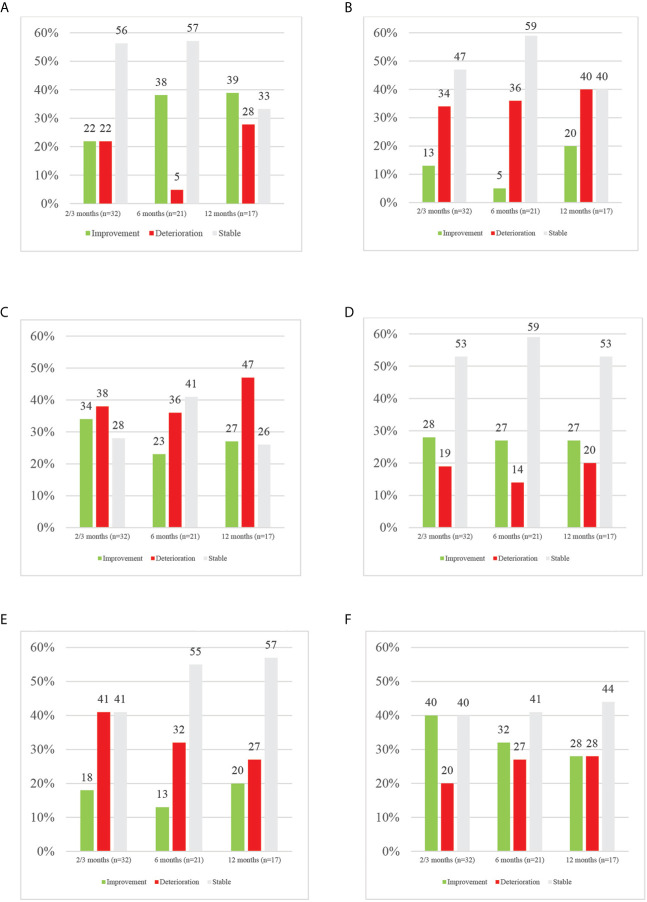
Overview of MCID in HRQoL and its domains per time point. **(A)** Overview of MCID in overall HRQoL per time point. **(B)** Overview of MCID in physical functioning per time point. **(C)** Overview of MCID in role functioning per time point. **(D)** Overview of MCID in emotional functioning per time point. **(E)** Overview of MCID in cognitive functioning per time point. **(F)** Overview of MCID in social functioning per time point.

**Figure 2** provides an overview of the percentages of MCID in overall HRQoL and its domains over time.

Overall HRQoL (p = .357) nor its domains (physical, p = .643; role, p = .069; emotional, p = .254; cognitive, p = 494; social, p = .735) did significantly change over time. In terms of MCID, throughout follow-up, the majority of patients remained stable in terms of overall HRQoL. At 2–3 months, an equal number (n = 7; 22%) of patients reported meaningful improved and deteriorated overall HRQoL. Half a year after end of treatment, a substantial percentage improved in HRQoL, whereas a very small number remained deteriorated (38% *vs* 5%). At one year after treatment, more of those patients still evaluable had a better overall HRQoL (39%) than baseline compared to deterioration (28%) or stable (33%) HRQoL. Among those with a meaningful clinical deterioration in overall HRQoL, at each time point, only one patient had progressive disease shortly after PRO data collection. More LA-NSCLC patients improved in overall HRQoL at 2–3 months (28% *vs* 15%) and 6 months (33% *vs* 14%) compared to metastatic patients. Those improving in the metastatic population were only those receiving immunotherapy, except for one patient at 12 months. It should however be noted that at 12 months, no patients receiving immunotherapy were still enrolled.

In contrast, focusing on the different domains of HRQoL, at any time point more patients deteriorated in physical, role and cognitive functioning than improved. In physical functioning, at 2–3, 6 and 12 months, deterioration was seen in 34, 36 and 40% of patients respectively compared to improvement in 13, 5 and 20% at the same time points. In the role domain, 38, 36 and 47% of patients deteriorated respectively at 2–3, 6 and 12 months while improvement was seen in 34, 23 and 27% of patients. In cognitive functioning, 41, 32 and 27% of patients experienced deterioration at the aforementioned time points, with 18, 13 and 20% improving. In contrast, in emotional and social functioning more improvements than deteriorations were reported, except for 12-months’ time point. Emotionally, 28, 27 and 22% of patients improved while 19, 14 and 20% deteriorated respectively at 2–3, 6 and 12 months. In the social domain, improvements were seen in 40% and 32% at 2–3 and 6 months, while deterioration was reported in 20% and 27% at abovementioned time points. At 12 months, an equal percentage of 28% deteriorated and improved in social functioning. In LA-NSCLC patients, improvements were dominating in role and social functioning; whereas in the metastatic population, mainly physical, emotional and cognitive functioning improved.

### Toxicity

**Figure 3:**
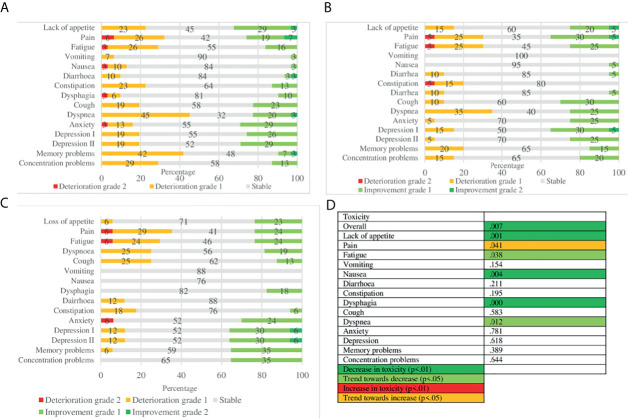
Toxicity over time: MCID and statistical significance. **(A)** MCID of toxicity 2/3 months after end of treatment. **(B)** MCID of toxicity 6 months after end of treatment. **(C)** MCID of toxicity 12 months after end of treatment. **(D)** Statistical significance.

**Figure 3** provides overviews of changes in toxicity over time, and its statistical significance. Overall toxicity decreased over time (p = .007). In terms of individual toxicities, lack of appetite (p = .001), nausea (p = .004) and dysphagia (p = .000) decreased over time. A trend towards an increase in pain (p = .041) is seen as well as a trend towards a decrease in fatigue (p = .038) and dyspnoea (p = .012). Most toxicities were short term, such as concentration and memory problems, dyspnoea, fatigue and pain. The most toxicity was reported 2–3 months after the end of treatment, particularly dyspnoea (45%), memory problems (42%) and pain (32%), with most improvements in lack of appetite (32%), anxiety (29%) and depression (28%). Overall most improvement in toxicity was reported at 6 months after treatment, mainly depression (35%), pain (35%) and cough (30%). In the LA-NSCLC population, most deteriorations were short term. However, dyspnoea remained deteriorating over time. Improvements are seen at the later endpoints in pain and cough. In metastatic patients, short-term deteriorations are mainly seen in pain, memory and concentration problems. Interestingly, at six months most notably are improvements in concentration problems (40%) and deteriorations in memory problems (40%).

### Neurocognitive Functioning

Overviews of statistical significance and MCID of NCF can be found in **Table 2**. Whereas in terms of statistical significance only positive associations and trends of NCF over time are found, especially in terms of visual memory and cognitive processing speed, specific neurocognitive domains are also negatively impacted, as can be observed in the MCIDs.

**Table 2:** Percentage of Minimally Clinical Important Difference (MCID) and statistical significance of neurocognitive tests over time.

Test		Minimally Clinical Important Difference (MCID) compared to baseline						Significance level
		2/3months		6 months		12 months		
		Deterioration	Improvement	Deterioration	Improvement	Deterioration	Improvement	
Verbal memory (HVLT-R test)		5 (17.9)	4 (14.2)	3 (15.0)	5 (25.0)	1 (5.9)	3 (17.6)	0.107
Verbal memory (HVLT-R test)		2 (8.3)	2 (8.3)	1 (5.0)	3 (15.0)	–	1 (5.8)	0.018
Visual memory (Reys complex test)		3 (11.1)	3 (11.1)	2 (10.5)	2 (10.5)	3 (17.6)	5 (29.4)	0
Visual memory (Reys complex test)		1 (4.0)	4 (16.0)	2 (10.5)	2 (10.5)	3 (17.6)	5 (29.4)	0
Recognition (HVLT-RT test)		–	–	–	–	–	–	0.999
Cognitive processing speed (TMT part A)		1 (3.6)	2 (7.1)	–	4 (20.0)	–	3 (17.6)	0
Executive functioning (TMT part B)		2 (8.3)	1 (7.4)	1 (5.0)	2 (10.0)	–	1 (5.9)	0.034
Executive functioning (Stroop test)		4 (15.4)	3 (13.0)	1 (5.3)	1 (5.3)	3 (17.6)	1 (5.9)	0.862
Word fluency (COWA)		1 (3.6)	1 (3.6)	–	–	1 (5.9)	–	0.014
Working memory (WAIS forward)		4 (15.4)	4 (15.4)	1 (5.0)	5 (25)	–	4 (23.5)	0.306
Working memory (WAIS backward)		1 (3.8)	–	–	1 (5.0)	–	2 (11.8)	0.085
Deterioration	Definition
	Deterioration between 10 and 19.9% of patients
	Deterioration in more than 20% of patients
Improvement	
	Improvement between 10 and 19.9% of patients
	Improvement in more than 20% of patients
Significance level	
	Positive correlation (p <.01)
	Trend towards positive correlation (p <.05)
	Negative correlation (p <.01)
	Trend towards negative correlation (p <.05)

Certain tests have missing data. MCID = 1 standard deviation.

Negative impact is particularly seen in working memory and executive functioning (at 2–3 months; 18 and 17% respectively) and in verbal memory (at 6 months; 15%). Visual memory deteriorated in a proportion of patients at each time, increasing at 12 months (17%).

Due to the limited data on NCF, particularly in the metastatic population, no analyses were conducted on the different disease stages.

## Discussion

This real-world (RW) study aimed to provide a synopsis of HRQoL, toxicity and NCF in patients with LA- and metastatic NSCLC receiving systemic and/or loco-regional radiotherapy.

The results demonstrated that long-term participation in the study was associated with better pre-treatment physical and role functioning, and a lower initial pain score. Among patients still participating at 1 year, more than one third had a meaningful improved overall HRQoL at this time point, despite potential treatment-induced toxicity. The entire population, certain pre-existing symptoms, such as lack of appetite, fatigue and dyspnoea, were palliated, while treatment-related toxicities (dysphagia, nausea) also decreased over time. For NCF, the overall tendency was towards improvement, especially the domains of visual memory and cognitive processing speed significantly improved over time.

Our data also suggest that LA- and metastatic NSCLC patients present with different baseline characteristics, which impacts their HRQoL. Of notice, more LA-NSCLC patients showed improved HRQoL compared to the metastatic population.

Physical and role functioning are known prognosticators for long-term survival in LA- and advanced NSCLC patients (23, 24). Physical health is also significantly correlated with HRQoL and cognitive functioning (25). These findings were confirmed in our study, observing a better pre-treatment physical and role functioning in patients who still participated at one year compared to those who stopped participation. Moreover, the long-term data on HRQoL and cognitive functioning in our study are mainly applicable to LA-NSCLC patients with better pre-treatment physical and role functioning, without progressive disease, thus experiencing longer overall survival without health deterioration.

In line with this, the fact that most of our patients had a meaningfully better HRQoL at one year after treatment than at baseline may be related to the LA-NSCLC patients dominating at 1 year. Also, treatment by itself seems to have a more positive impact on HRQoL in the group of LA-NSCLC patients compared to metastatic patients. This may be partially explained by the fact that LA-NSCLC patients receive treatment with curative intent, whereas treatment for metastatic NSCLC aims to control disease and palliate symptoms rather than to cure. The notion that no cure is available, causes more hopelessness in those receiving palliative treatment, consequently potentially impacting HRQoL negatively (26). While it is known that chemotherapy has a positive impact on HRQoL and pre-existing symptoms in advanced NSCLC patients (27), this explanation hardly applies to our population, as unfortunately almost no metastatic patient survived at one year. Improvements in HRQoL may also be partially explained by a so-called ‘response shift’. Patients’ internal standards, values and conceptualization of HRQoL may change over the course of the disease trajectory (28). Patients may gradually accept the problems impacting their HRQoL and thus no longer report this as an issue for their HRQoL.

Conversely, at the earlier evaluation point of 2–3 months post-treatment, more meaningful deteriorations than improvements in HRQoL were reported. Similar results were found in a study on the impact of radiotherapy with or without chemotherapy in NSCLC patients (29). This may seem surprising, as at that moment most acute side-effects should have resolved.

In our study, most toxicities were acute and eventually improved over time. Acute dyspnoea and memory problems were the most prominent short-term toxicities.

Acute induced dyspnoea is consistent with the radio-pneumonitis typically occurring within the first month post-radiotherapy (30) and chemotherapy-induced injury to respiratory muscles and the peripheral nervous system (31). Still, it should be noted that in the LA-NSCLC patients, dyspnoea remained impacted over time.

Memory loss may be caused by fatigue, anxiety and depression resulting from treatment as well as chemotherapy-induced cognitive changes (32). Treatment-induced fatigue, on top of pre-existing fatigue, was experienced in 29% of patients at that time point and may therefore have contributed to memory problems. It can be hypothesized that deterioration in memory loss as a result of depression and anxiety at 2–3 months may result from less intensive follow-up compared to during and immediately after treatment, which may cause distress (33, 34). Furthermore, this is the moment of the first computerized tomography (CT) scan after the end of treatment, potentially causing so-called ‘scanxiety’ (33). However, our study found that only a limited number of patients, 6 and 12% respectively, had a meaningful worsening in anxiety and depression at 2–3 months. Moreover, of interest is that memory and concentration problems improved importantly at 6 months in the metastatic patient population.

While therapy causes toxicity, it also may have a positive impact on pre-existing symptoms. In our study, baseline lack of appetite, anxiety and depression improved the most at 2–3 months. The increase in appetite may be explained by the high anxiety levels at diagnosis, which reduces the motivation for food intake and pleasure of eating. The initial decrease in anxiety, persisting over time, may have resulted in improved appetite. The decrease in anxiety and depression at the end of chemotherapy, although not returning to baseline, has been previously found in lung cancer patients (35). An explanation could be that patients develop constructive strategies to deal with their diagnosis and lung cancer-related stigma. Furthermore, treatment may bring hope and optimism, alleviating psychological symptoms (36).

In addition, fatigue and dyspnoea, frequent pre-existing symptoms in lung cancer patients, due to the disease itself or because of co-morbidity, tended to improve over time in our population.

It is not easy to disentangle pre-existing cancer-related symptoms from treatment related toxicities. Therefore, in this study we both evaluated the results in terms of statistical significance and individual changes over time. Statistical significance levels provide hypotheses on differences between averages of groups or time points, whereas individual meaningful evolutions over time allow to identify the magnitude of those positively and negatively affected by therapy (37–39). The latter is commonly applied to HRQoL by defining MCIDs, which are widely accepted and recommended, with a 10 point change from baseline being accepted as clinically relevant (39). Similarly, in line with previous research on NCF, MCID in NCF tests was set at one standard deviation difference between baseline and each subsequent time point (40–43).

In the context of toxicity, improvements and deteriorations were calculated by taking the baseline scores into account. This has, indeed, a major advantage as in that it allows to better make the distinction between treatment-related toxicity and the response to therapy of pre-existing symptoms at an individual level (30). This especially applies to some of the toxicities evaluated in this study, such as dyspnoea or fatigue: these can already be present at baseline, in which case they would better be denoted as ‘symptoms’ rather than ‘toxicity’. A decrease of dyspnoea, for instance, would then be a symptomatic improvement, whereas an increase in dyspnoea may both be the result of treatment toxicity as well as a reflection of symptomatic worsening. De Ruysscher et al. have also applied this method in evaluating the impact of high-dose radiotherapy on dyspnoea among NSCLC patients (30). Their data showed that dyspnoea, indeed, could improve and worsen over time, may be acute and be resolved within weeks or may protract over time with patients remaining dyspneic.

Our study is one of the first to evaluate NCF in lung cancer patients without local treatment to the brain. An interesting finding was that NCF tends to significantly improve over time. A recently published review also showed an improvement in attention over the course of chemotherapy (6). This may be due to the ‘practice effect’, which refers to improvements in NCF test performance due to repeated evaluation of the same test (44).

On the other hand, meaningful clinical deteriorations in certain neurocognitive domains, particularly at 2–3 but up to 6 months after treatment, were found. This is in line with findings of a pilot study on NCF in LA-NSCLC patients receiving chemo-radiotherapy: NSCLC patients exhibited cognitive dysfunction one month post-treatment, but recovered at 7 months (45). Other research also showed that long-term neurocognitive deficits were minimal (6). While NCF data of lung cancer patients being treated with chemotherapy—all or not in conjunction with loco-regional radiotherapy—remain limited, the available evidence thus seems to suggest that neurocognitive decline might be temporary. Research on patients with brain metastases receiving whole-brain radiotherapy (WBRT), conversely, shows that a decline in NCF over time, particularly in memory and executive functioning, leads subsequently to impaired HRQoL (46).

The strengths of this study are the collection of both HRQoL and toxicity data with PROMs. Toxicity is mostly clinician-scored, however, patients are experts in their health status and clinical problems. PROMs therefore assess patients’ wellbeing and symptoms more objectively than clinicians (47). Clinicians tend to underreport symptoms and problems and focus on what they expect.

Moreover, this study collected RW data to reflect on real clinical outcomes of NSCLC patients receiving standard treatment outside of the context of randomized controlled trials. Data from randomized trials may lack external validity, since typically less than 5% of cancer patients are enrolled in trials, even though 70% of patients report to be willing to participate (48). In the context of lung cancer, particularly patients with poor performance status, multiple co-morbidities, brain metastases, rare oncogenic driver mutations or elderly, are often excluded (49). This study did not exclude patients based on aforementioned criteria in order to provide data on the heterogeneous group of lung cancer patients seen in the clinic.

Another strength is the comprehensive data collection with PROMs and NCF tests to explore psychological, cognitive, physical and social wellbeing of patients in current practice. Data is limited on NCF in NSCLC patients. The majority of studies focus on patients treated with prophylactic cranial or WBRT whereas the impact of loco-regional radiotherapy or systemic therapy has rarely been studied (45, 50–53).

Naturally, the current study also has a number of limitations. Firstly, the small sample of 50 patients was recruited from one single academic hospital in Belgium, which could compromise the generalizability of the findings. Therefore, this study can only be seen as exploratory.

There was also a significant patient-drop out, mainly due to death and deterioration in health. Missing data is a common phenomenon in observational lung cancer studies with serial measurement designs (54, 55). Missing data is particularly encountered in metastatic patients, hence may not be at random. Also, patients with poor pre-treatment health and HRQoL are more likely to discontinue participation. Besides death, follow-up terminated in our study due to changes in therapy or the patients’ decision to not further participate. Of the 24 patients alive at 1 year in our study; 18 (75%) were still participating. Of these, 15 (or 58% of the original 26), were from the LA-NSCLC population. This participation rate is deemed reasonable for a lung cancer trial (56). As a matter of comparison, in a dose-escalation study on LA-NSCLC patients, 57% of living patients and 44% of all patients completed PROMs at 12 months. This was considered an appropriate compliance rate. Moreover, statistical methods dealing with missing data, essential to ensure internal validity and generalizability, were used (37).

Lastly, analysis was hampered by the different measurement time points between LA- and metastatic patients, reflecting actual clinical practice.

Due to the limitations of this study, particularly the small sample size and mainly descriptive statistics applied, the results should be seen as explorative. Therefore firm conclusions are hard to draw.

In conclusion, despite the fact that our study did not show any significant differences over time in overall HRQoL or its domains in the total population, a proportion of patients experienced clinically meaningful improvements as well as deteriorations. Long-term HRQoL meaningfully improved in more than one-third of patients still alive without progressive disease at one year post-treatment, notwithstanding treatment-induced toxicities. LA- and metastatic patients present different baseline characteristics, subsequently impacting HRQoL. More LA-NSCLC patients showed improved HRQoL compared to metastatic patients.

Toxicities typically resolved within the first months, whereas certain pre-existing symptoms, particularly dyspnoea and neuro-psychological symptoms were alleviated. While some patients experienced meaningful cognitive impairment in some domains of NCF, the overall tendency was towards improved NCF. As LA- and metastatic NSCLC patients have poor prognoses, it is important to understand the impact of treatment on HRQoL, symptoms and NCF for each individual patient to aid decision-making regarding quality and quantity of life.

## Data Availability Statement

The raw data supporting the conclusions of this article will be made available by the authors, without undue reservation.

## Ethics Statement

The studies involving human participants were reviewed and approved by the Ethical Committee Ghent University Hospital. The patients/participants provided their written informed consent to participate in this study.

## Author Contributions

All authors contributed to conception and design of the study. LW collected data and performed the statistical analyses. All authors contributed to the article and approved the submitted version.

## Conflict of Interest

The authors declare that the research was conducted in the absence of any commercial or financial relationships that could be construed as a potential conflict of interest.
